# Roseotoxin B alleviates cholestatic liver fibrosis through inhibiting PDGF-B/PDGFR-β pathway in hepatic stellate cells

**DOI:** 10.1038/s41419-020-2575-0

**Published:** 2020-06-15

**Authors:** Xingqi Wang, Yuzhi Gao, Yu Li, Yuqing Huang, Yawen Zhu, Wei lv, Ruzeng Wang, Lingshan Gou, Chao Cheng, Zhaojun Feng, Jun Xie, Jun Tian, Ruiqin Yao

**Affiliations:** 1 Key Laboratory for Biotechnology on Medicinal Plants of Jiangsu Province, School of Life Science, Jiangsu Normal University, 101 Shanghai Road, Xuzhou, 221116 Jiangsu China; 2 Department of Cell Biology, Jiangsu Key Laboratory of New Drug Research and Clinical Pharmacy, Xuzhou Medical University, 209 Tongshan Road, Xuzhou, 221009 Jiangsu China; 3 Center for Genetic Medicine, Xuzhou Maternity and Child Health Care Hospital, Xuzhou, 221009 Jiangsu China

**Keywords:** Pharmacology, Experimental models of disease

## Abstract

Identifying effective anti-fibrotic therapies is a major clinical need that remains unmet. In the present study, roseotoxin B was shown to possess an improving effect on cholestatic liver fibrosis in bile duct–ligated mice, as proved by histochemical and immunohistochemical staining, hepatic biochemical parameters, and TUNEL apoptotic cell detection in tissue sections. Using cellular thermal shift assay, computational molecular docking, microscale thermophoresis technology, and surface plasmon resonance biosensor, we confirmed that PDGFR-β was a direct target of roseotoxin B in fibrotic livers. Of note, human tissue microarrays detected pathologically high expression of p-PDGFR-β in liver samples of ~80% of patients with liver fibrosis and cirrhosis. PDGF-B/PDGFR-β pathway promotes transdifferentiation and excessive proliferation of hepatic stellate cells (HSCs), which is a very crucial driver for liver fibrosis. Meaningfully, roseotoxin B blocked the formation of PDGF-BB/PDGFR-ββ complex by targeting the D2 domain of PDGFR-β, thereby inhibiting the PDGF-B/PDGFR-β pathway in HSCs. In summary, our study provided roseotoxin B as a unique candidate agent for the treatment of liver fibrosis.

## Introduction

In the process of chronic or iterative wound healing response to long-standing liver damage, such as cholestatic liver disease, livers often tend to develop into hepatic fibrosis which is a final common adverse consequence^1,2^. However, if the chronic liver condition is not well controlled, it may progress to more terrible consequences^1,2^. Actually, ~80% of primary liver malignancies develop on a fibrotic or cirrhotic background^3–5^. Therefore, liver fibrosis is a major risk factor to develop hepatocellular carcinoma that is already the fifth most common solid malignancy and the third leading cause for cancer-related death^3–5^. It can be seen that liver fibrosis is seriously threatening the health and life of human beings worldwide. We should actively explore effective therapeutic methods to alleviate liver fibrosis.

Targeting the underlying cause of liver fibrosis is a very important therapeutic strategy to effectively attenuate this disease^6,7^. At present, multiple medical approaches have been used in clinical treatment to stabilize the progression of liver fibrosis, such as abstinence from alcohol in alcoholic liver disease, lifestyle changes and weight control in fatty liver disease, antiviral treatment in hepatitis B virus infection^6,7^. However, it is still extremely necessary to develop more types of anti-fibrotic drugs to treat liver fibrosis. There is convincing evidence to prove that transdifferentiated (or activated) hepatic stellate cell (HSC) is the major driver of liver fibrogenesis^8,9^. In a normal liver, HSCs are quiescent, and they are mainly located in the perisinusoidal space between sinusoidal endothelial cells and hepatocytes, mostly function as vitamin A reserves^8^. However, in response to chronic liver injury, paracrine signals and inflammatory mediators from fibrotic tissue microenvironment promote HSCs activation and differentiation into the major cellular source of matrix protein-secreting myofibroblasts^8,9^. Activated HSCs account for ~80% of total fbrillar type I collagen in the fibrotic liver^8^. Therefore, a therapeutically valuable compound that could induce the depletion or deactivation of the activated HSCs is critical for the resolution of liver fibrosis.

As early as 1975, roseotoxin B had been identified^10^. However, studies on the pharmacological activity of roseotoxin B has been very rare so far. Specifically, it mainly includes the following findings: a research had shown that roseotoxin B can cause a significant mortality to fall armyworm and corn earworm^11^; two studies had found that roseotoxin B can produce positive inotropic and negative chronotropic effects on cardiac functions, indicating that roseotoxin B may assist the therapy of heart failure without aggravating the pathophysiological changes in the failed heart^12,13^; additionally, our previous study revealed that roseotoxin B can ameliorate allergic contact dermatitis^14^. In the present study, we discovered another novel pharmacological activity of roseotoxin B, which could improve cholestatic liver fibrosis in mice via blocking PDGF-B/PDGFR-β pathway in HSCs. There is sufficient evidence now that HSCs are the main cellular target for the treatment of liver fibrosis, and the dysregulated PDGF-B/PDGFR-β signaling pathway is an important inducement for the pathological transdifferentiation of HSCs and crucial pathological factor for liver fibrosis^9,15,16^. Therefore, the pharmacological intervention of roseotoxin B to therapeutically restrain activated HSCs would enable an effective anti-fibrotic strategy.

## Materials and methods

### Mice, cell, and reagents

C57BL/6 mice (female, 20–22 g, and 8-week old) were obtained from the Model Animal Genetics Research Center of Nanjing University (Nanjing, China). Mice were assigned randomly to five groups, and they were maintained with free access to water and pellet food in plastic feeding cages at 21 ± 2 °C and kept on a 12 h dark/light cycle in specific-pathogen-free (SPF) facilities. Experimental procedures and animal welfare were carried out strictly in accordance with the Guide for the Care and Use of Laboratory Animals (National Institutes of Health, the United States; Order No. 1998-55, Ministry of Public Health, China) and related ethical regulations of our universities. All animal experiments were approved by the Animal Care and Use Committee of our universities and made to minimize suffering and to reduce the number of animals used. No animals were excluded from the analysis. No blinding was carried out for animal experiments, but data analysts were blinded to grouping. CFSC-8B cell and LX-2 cell were purchased from Shanghai Institute of Cell Biology (Shanghai, China). The cell lines are authenticated by STR profiling, and they are not contaminated with mycoplasma.

Roseotoxin B (99% of purity) was dissolved at a concentration of 50 mM in DMSO as a stock solution, and diluted with medium before each experiment. The final DMSO concentration in each group did not exceed 1‰ throughout the study. Crystal violet and MTT (3-(4, 5-dimethyl-2-thiazyl)-2, 5-diphenyl-2H-tetrazolium bromide) were purchased from Sigma-Aldrich (St Louis, MO). Amine-coupling kit and CM5 sensor chips were obtained from General Electric Company (Shanghai, China). Annexin V-FITC and propidium iodide (PI) were purchased from BD Biosciences (San Jose, CA). Antibodies against Akt (#4691), p-Akt (#4060), PDGFR-β (#3169), ERK (#4695), p-ERK (#8544), Cyclin D1 (#2978), Caspase-3 (#9662), and p-Rb (#8180) were purchased from Cell Signal Technology (Beverly, MA). Antibodies against β-actin (sc-47778), GAPDH (sc-365062), Col1A1 (sc-293182), Col3A1 (sc-271249), and α-SMA (sc-53142) were purchased from Santa Cruz Biotechnology (Santa Cruz, CA). Antibodies against Rb (ab226979) and p-PDGFR-β (ab16868) were purchased from Epitomics (Burlingame, CA). Recombinant human PDGFR-β protein (His tag) was purchased from Sino Biological (Beijing, China). Bilirubin assay kit, ALT assay kit, AST assay kit, hydroxyproline assay kit, and Masson staining assay were obtained from Nanjing Jiancheng Bioengineering Institute (Nanjing, China). Human PDGFR-β kinase activity kits were from R&D Systems (Minneapolis, MN). Hematoxylin and Eosin (H&E) staining kit and fast silver stain kit were purchased from Beyotime Biotechnology (Nantong, China). All other chemicals were purchased from SigmaAldrich (St Louis, MO). Tissue microarray was obtained from Alenabio Biological Technology Co., Ltd (Xian, China), and all human tissues are collected under IRB and HIPPA approved protocols.

### Cell culture and cell proliferation assay

The rat hepatic stellate cell (HSC) line CFSC-8B and immortalized human HSC line LX-2 were maintained in plastic culture dishes in Dulbecco’s modified Eagle’s medium (DMEM) supplemented with 10% fetal bovine serum (FBS), 100 μg/ml of streptomycin, and 100 U/ml of penicillin under a humidified 5% (v/v) CO_2_ atmosphere at 37 °C. Cell proliferation was determined by MTT assay.

### Western blot

This work was performed as our previously reported^17^. Proteins were extracted in lysis buffer. The proteins (10–30 μg per lane) were then separated by SDS–PAGE (10%) and electrophoretically transferred onto polyvinylidene fluoride membranes. The membranes were probed with antibodies overnight at 4 ^o^C, and then incubated with a HRP-coupled secondary antibody. Detection was performed using a LumiGLO chemiluminescent substrate system (KPL, Guildford, UK).

### Surface plasmon resonance (SPR)

This work was performed as our previously reported^18^. Briefly, PDGFR-β was immobilized on a Biacore CM5 sensor chip via the primary amine groups. Roseotoxin B solution flowed at a rate of 30 μl/min for 60–80 s to allow for association, followed by 200 s for dissociation over immobilized protein in PBS running buffer (pH 7.4, 0.5% P20 surfactant, 1.05 × PBS). Roseotoxin B was tested for binding at 3.125–100 μM. Normalization of the data involved transformation of the *y*-axis such that the theoretical maximum amount of binding for a 1:1 interaction with the protein surface corresponded to a sensor response of 100 relative units (RU).

### HSC migration assay

Specifically, confluent CFSC-8B cells at the top of BIOCOAT MATRIGEL invasion chambers were incubated in serum-free medium for 24 h. The lower chamber was filled with PDGF-BB (10 ng/ml) in the presence or absence of roseotoxin B at incremental concentrations. They were incubated for 24 h, and the CFSC-8B cells from the upper surface of the membranes were then completely removed with gentle wiping. The migrated cells on the lower surface of the membranes were fixed and stained with crystal violet. The migration rate of these cells was determined by counting the number of stained cells on the membranes in six randomly selected fields.

### HSC wound healing assay

CFSC-8B cells were seeded in 6-well plates and grown to confluence in DMEM containing 10% FBS. Confluent CFSC-8B cells were deprived of serum for 24 h and then disrupted to generate a linear wound, followed by incubation in DMEM containing PDGF-BB (10 ng/ml) in the presence or absence of roseotoxin B for 48 h. They were subsequently fixed and observed under phase-contrast microscopy, and six randomly selected points of each well along each wound were measured.

### Immunohistochemistry

Paraffin-embedded liver tissue sections were heat-fixed, blocked with 3% H_2_O_2_, and incubated with specific antibodies (1:100 diluted) overnight at 4 °C. Detection was done using the staining kits from ThermoFisher SCIENTIFIC (Shanghai, China) according to their protocols for immunohistochemistry.

### TUNEL assay

TUNEL bright green apoptosis detection kit was obtained from Vazyme (Nanjing, China), and apoptotic cells in the liver tissue sections were detected by this kit according to the manufacturer’s instructions. Images were observed under a fluorescence microscope (Olympus, Tokyo, Japan).

### Cell apoptosis assay

LX-2 cells were maintained in DMEM supplemented with PDGF-BB (10 ng/ml) in the presence or absence of roseotoxin B for 24 h. Cell apoptosis was measured by flow cytometry after addition of PI and FITC-conjugated annexin V assay. These cells were analyzed by the flow cytrometry on a FACScan (Becton Dickinson). Data were analyzed with CELLQuest software (BD Biosciences).

### Cell cycle assay

LX-2 cells were maintained in DMEM supplemented with PDGF-BB (10 ng/ml) in the presence or absence of roseotoxin B for 24 h, and they were collected in cold PBS (4 °C) then fixed with ethanol (70%) at 4 °C overnight. Afterward, these fixed cells were washed with cold PBS (4 °C) and stained with PI (50 μg/ml) containing 1% TritonX-100 and 100 μg/ml of RNase A at room temperature in the dark for 45 min. The DNA contents of these cells were analyzed with Modfit software (Becton Dickinson, San Jose, CA, USA).

### Liver fibrosis induced by ligation of the common bile duct (BDL)

For murine liver fibrosis induced by BDL study, mice at 8–10 weeks of age were anesthetized (*n* = 8 in each group). After midline laparotomy, the common bile duct was double-ligated and transected between the ligatures. The sham control group was similarly subjected to the operation without BDL. After BDL for 6 days, roseotoxin B (5–10 mg/kg/d) was administered for another 8 days. Mice that underwent BDL for 6 and 14 days served as model controls for the roseotoxin B treatment.

### Cellular thermal shift assay (CESTA)

This work was performed as previously reported^19,20^. Proteins were extracted from BDL-induced fibrotic liver tissue, and protein solution was equally divided into two groups including DMSO-incubated group and roseotoxin B-incubated group. The protein solution was incubated with DMSO or roseotoxin B (10 μM) for 3 h, then proteins of these two groups were subjected to CESTA. Briefly, incubated protein solution was equally divided into ten parts, each part got heated for 3 min under different temperatures (70, 67, 64, 61, 58, 55, 52, 49, 46, 43 °C). After that, the fibrotic liver tissue lysates were extracted by centrifugation at 20,000 × *g* for 20 min. Proteins expression was detected by SDS/PAGE and silver stain analysis, and the screened proteins were identified using LC/MS analysis.

### Statistical analyses

Data analysts were blinded to grouping. No data was excluded from analysis. Statistical tests for every figure are justified as appropriate. In order to choose the sample size, power analysis was performed according to previous report^21^. The result demonstrated that a sample size of 4–6 mice/group for evaluations of liver fibrosis in mice will provide at least 80% power (1-β) for data analysis at a 0.05 alpha level. All results shown represent means ± SEM from triplicate experiments performed in a parallel manner. Statistical analysis was performed with GraphPad Prism^®^ software (Version 8.1.1, San Diego, CA, USA). The variance similar between the groups had been statistically compared. Data were statistically evaluated by one-way ANOVA followed by Dunnett’s test between control group and multiple dose groups. The level of significance was set at a *P* value of 0.05.

## Results

### Roseotoxin B therapeutically improved bile duct ligation (BDL)-induced liver fibrosis in vivo

**Fig. 1:**
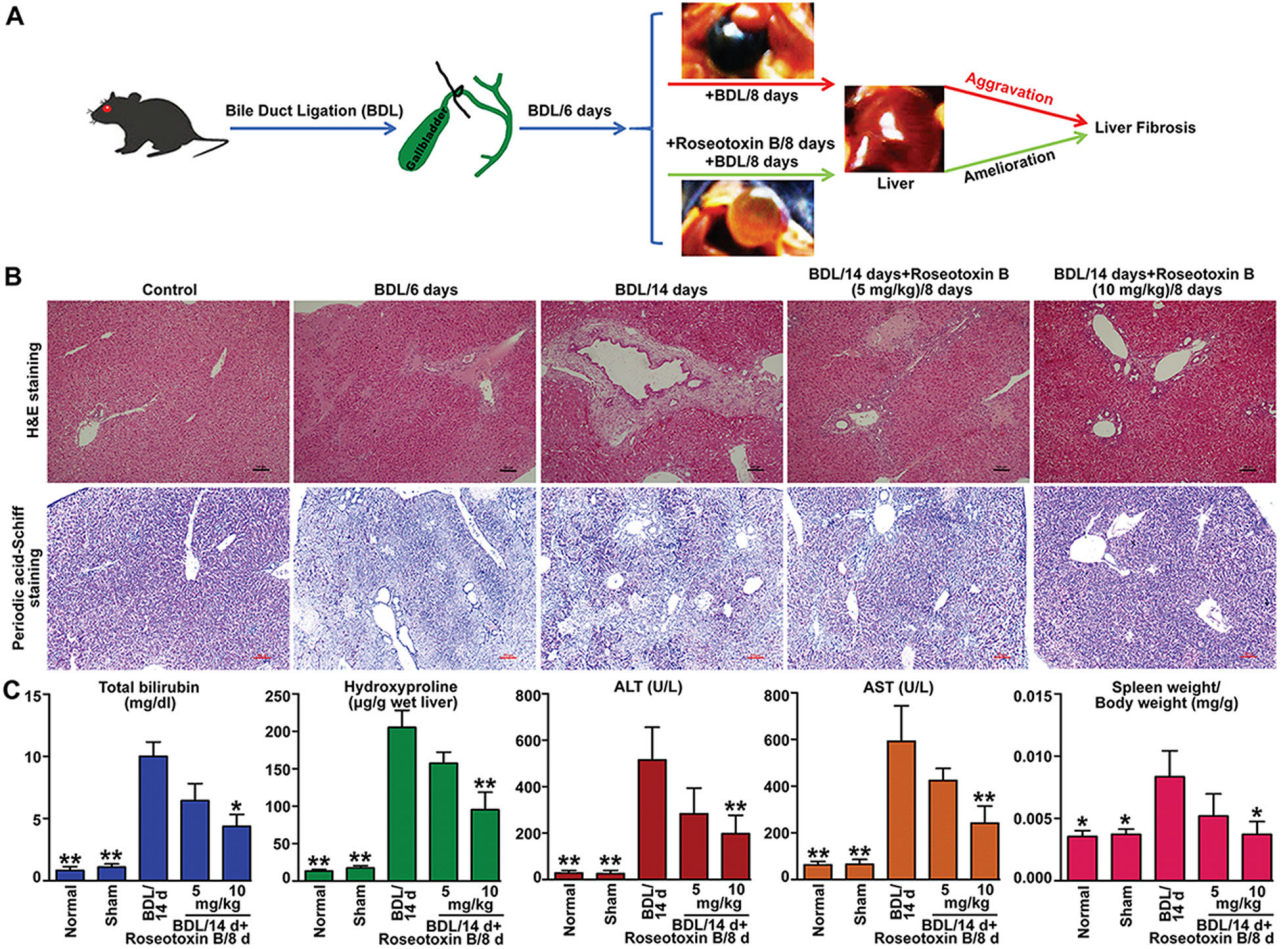
Roseotoxin B extenuated BDL-induced liver damage and fibrosis in mice. Liver fibrosis was induced by BDL operation. After BDL for 6 days, roseotoxin B was administered at 5–10 mg/kg/day for another 8 days (*n* = 8 in each group). **a** Overview of the in vivo experiment. **b** Effects of roseotoxin B on liver injury and fibrosis in BDL mice. Sections of murine liver were stained with hematoxylin–eosin (H&E) staining and periodic acid-Schiff staining, which were examined by a blinded histologist (scale bar = 100 μm). **c** The total bilirubin content in serum, liver hydroxyproline content, ALT content in serum, AST content in serum and spleen index were tested in this study. The data are expressed as histograms illustrating means ± SEM of three independent experiments. ^*^P < 0.05, ^**^P < 0.01 versus the BDL/14 day group.

**Fig. 2:**
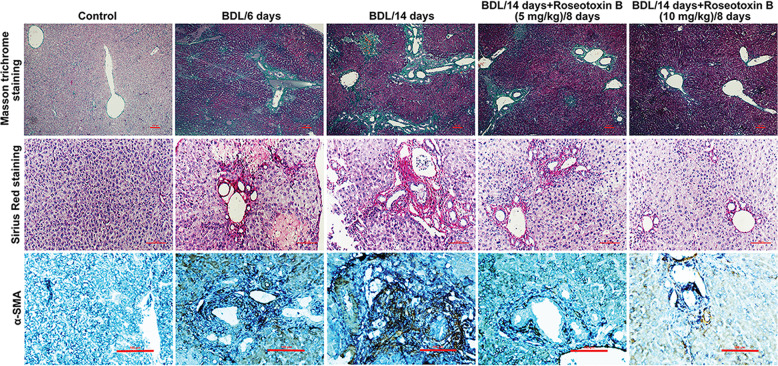
Roseotoxin B mitigated BDL-induced liver fibrosis in mice (*n* = 8 in each group). Effect of roseotoxin B on collagen expression in mice with cholestatic liver fibrosis. Sections of murine liver were stained with Masson trichrome staining and Sirius Red staining, which were examined by a blinded histologist (scale bar = 100 μm); hepatic immunohistochemical staining was used to evaluated the effect of roseotoxin B on α-SMA expression in mice with cholestatic liver fibrosis (scale bar = 100 μm). The data represented here represent one of three independent experiments with similar results.

**Fig. 3:**
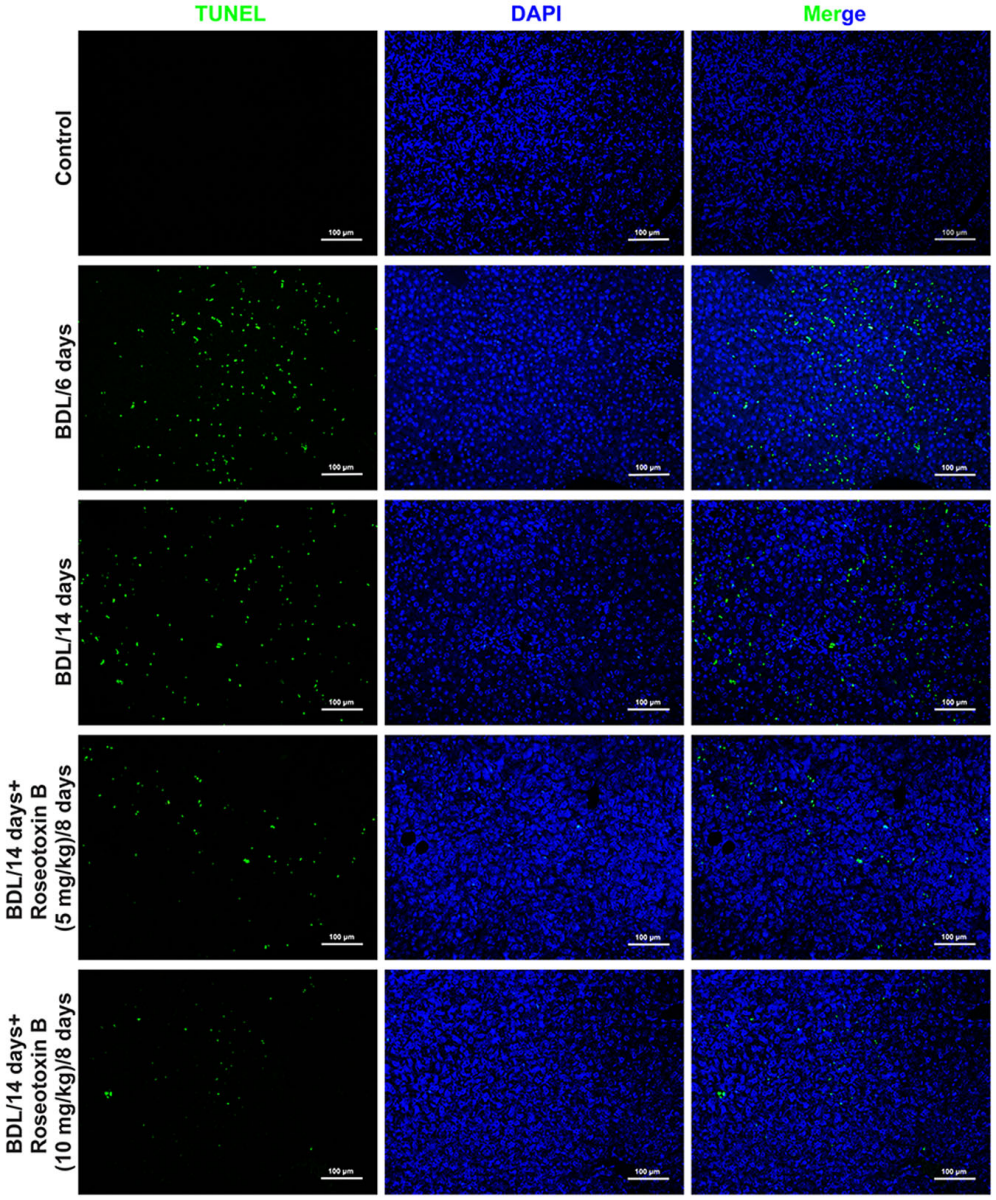
Roseotoxin B attenuated BDL-induced hepatocyte apoptosis in non-fibrotic regions of experimental fibrotic liver tissue. Sections of murine liver were stained for TUNEL (green) and DAPI (blue) (scale bar = 100 μm). The data represented here represent one of three independent experiments with similar results.

In the present study, we used a therapeutic administered model (administration of roseotoxin B from day 7 to day 14) to investigate the protective effect of roseotoxin B on BDL-induced cholestatic liver fibrosis (Fig. 1a). As shown in Fig. 1b, six days after BDL surgical operation, liver injury and fibrosis were observed in BDL-treated mice, including enlargement of the portal area, increased number of small bile ducts, mild fibrous hyperplasia, mild to moderate focal necrosis of hepatocytes, and reduced cellular glycogen storage. These pathological changes became more severe in BDL/14 days-group (Fig. 1b). However, the therapeutic administration of roseotoxin B obviously improved liver damage, relieved fibrosis accumulation, and maintained hepatocellular glycogen stores (Fig. 1b). Additionally, we evaluated serum total bilirubin, hydroxyproline content, serum aspartate aminotransferase (AST) and alanine aminotransferase (ALT) levels and spleen weight/body weight ratio to provide evidence of the protective effect and anti-fibrotic properties of roseotoxin B in BDL-induced liver fibrosis (Fig. 1c). As shown in Fig. 1c, the levels of ALT, AST and serum total bilirubin, the hydroxyproline content, and the spleen values were evidently decreased in mice with liver fibrosis following therapeutic treatment with roseotoxin B. The anti-fibrotic properties of roseotoxin B was further verified by Masson trichrome staining, Sirius Red staining, and α-SMA tissue expression (Fig. 2 and Supplementary Fig. 1). Roseotoxin B dramatically mitigated the levels of tissue collagen content, fibrous hyperplasia and α-SMA expression in BDL-induced liver fibrosis (Fig. 2 and Supplementary Fig. 1). BDL-induced liver fibrosis is accompanied by hepatocytes apoptosis which is an important feature of liver injury^22,23^. However, the number of apoptotic cells marked by TUNEL fluorescence staining in non-fibrotic regions of the fibrotic liver tissue was evidently decreased in following therapeutic treatment with roseotoxin B (Fig. 3 and Supplementary Fig. 2A).

### PDGFR-β may be a direct target protein for roseotoxin B in fibrotic liver

Given that roseotoxin B improved liver fibrosis (Figs. 1 and 2), we would like to know more about the direct target of roseotoxin B in fibrotic liver. It is well known that CETSA can be used to evaluate drug target interactions in cells and monitor drug target engagement in cells and tissues^19,20^. In this study, the CETSA, SDS/PAGE, silver stain analysis, and LC/MS analysis were used to screen out the potential direct target protein (or proteins) of roseotoxin B in BDL-induced fibrotic liver. As shown in the dashed boxes, roseotoxin B-induced stabilization of protein expression was investigated and the LC/MS analysis demonstrated that PDGFR-β was a target protein of roseotoxin B in fibrotic liver (Supplementary Fig. 3).

### Phosphorylated PDGFR-β was highly expressed in livers of human patients with viral hepatitis-inflammation, cirrhosis, and hepatocellular carcinoma

**Fig. 4:**
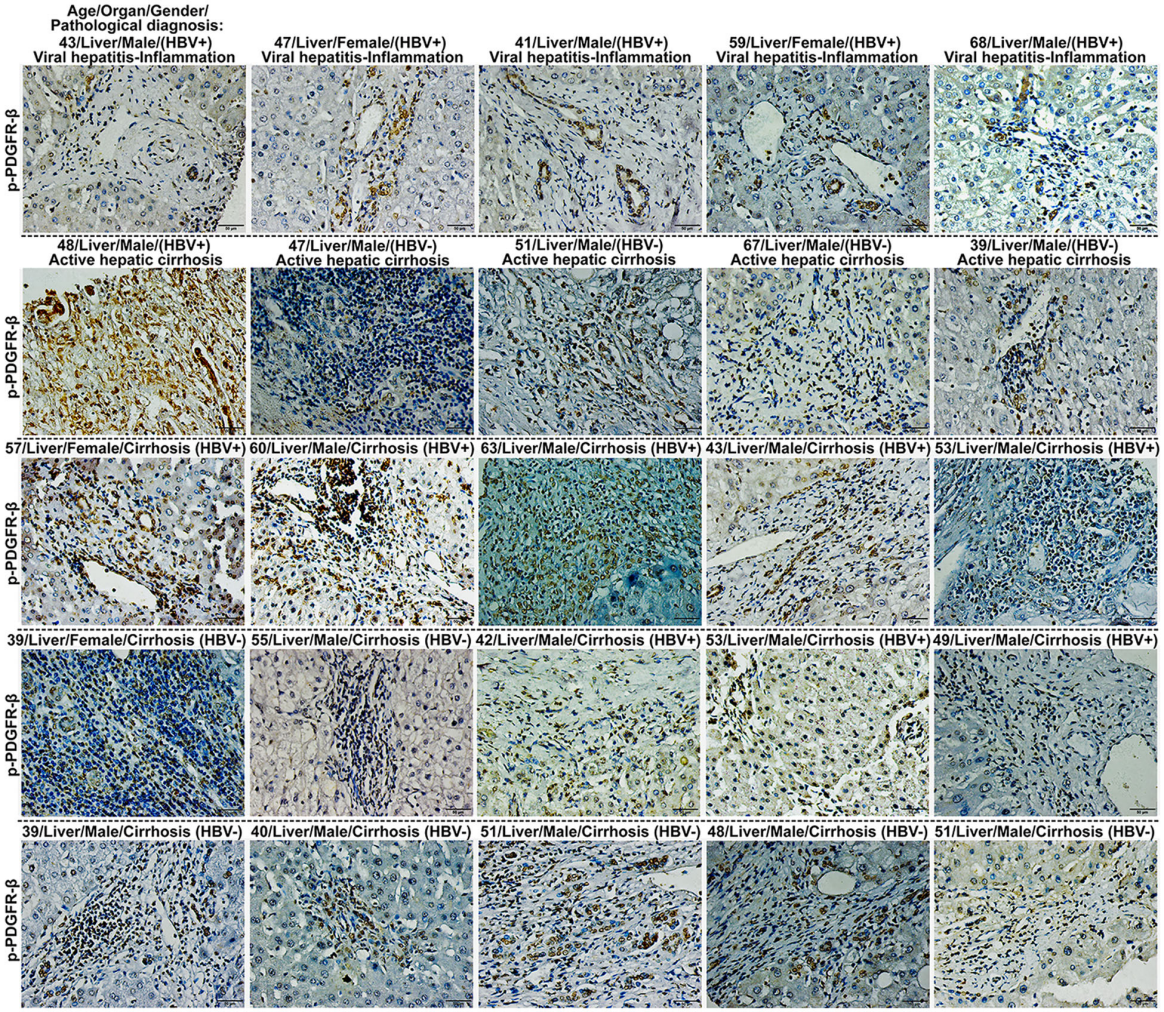
Phosphorylated PDGFR-β was highly expressed in about 80% of human liver tissues with viral hepatitis-inflammation and cirrhosis. The expression of p-PDGFR-β in liver samples with viral hepatitis-inflammation from 10 HBV-positive patients, and cirrhotic liver samples from 62 patients was assessed using tissue microarray. Hepatic immunohistochemical staining was performed on paraffin sections of human liver tissue with p-PDGFR-β antibody (scale bar = 50 μm).

**Fig. 5:**
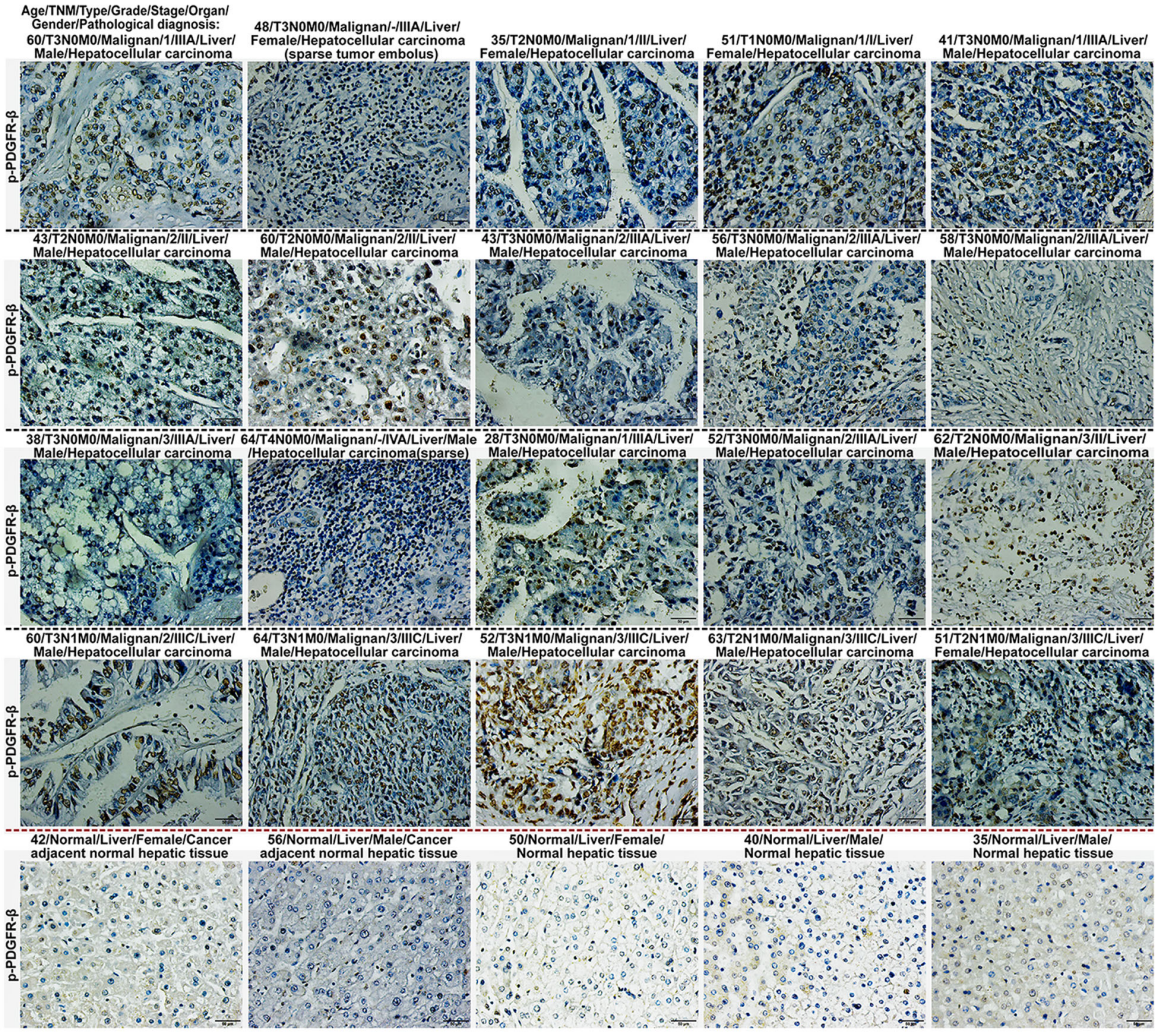
Phosphorylated PDGFR-β was highly expressed in about 39% of human liver tissues with hepatocellular carcinoma. The expression of p-PDGFR-β in liver samples with hepatocellular carcinoma from 54 HBV-positive patients and 35 normal controls was assessed using tissue microarray. Hepatic immunohistochemical staining was performed on paraffin sections of human liver tissue with p-PDGFR-β antibody (scale bar = 50 μm).

To test whether p-PDGFR-β overexpression is observed in hepatic tissue of human patients with chronic liver disease, 161 liver samples including 10 samples with viral hepatitis-inflammation, 62 cirrhotic liver samples and 54 hepatocellular carcinoma tissue samples (HBV-positive) were analyzed using immunohistochemistry in this study (Figs. 4 and 5). The results revealed that eight patients (Positive rate = 0.80, 8/10) with viral hepatitis-inflammation and 50 patients (Positive rate = 0.81, 50/62) with cirrhosis showed excessive expression level of p-PDGFR-β in the liver fibrotic areas (Supplementary Fig. 2B), while 21 patients (Positive rate = 0.39, 21/54) with hepatocellular carcinoma showed high expression of p-PDGFR-β in their liver samples (Supplementary Fig. 2B), whereas the hepatic tissue samples in normal controls (*n* = 35) barely expressed visible p-PDGFR-β (Supplementary Fig. 2B). Further analysis of the non-cancerous liver samples, we found that 65.9% (27/41) of HBV-positive patients and 74.2% (23/31) of HBV-negative patients exhibited overexpression of p-PDGFR-β in the fibrotic area of their liver samples.

### Roseotoxin B inhibited PDGF-B/PDGFR-β pathway in HSCs

**Fig. 6:**
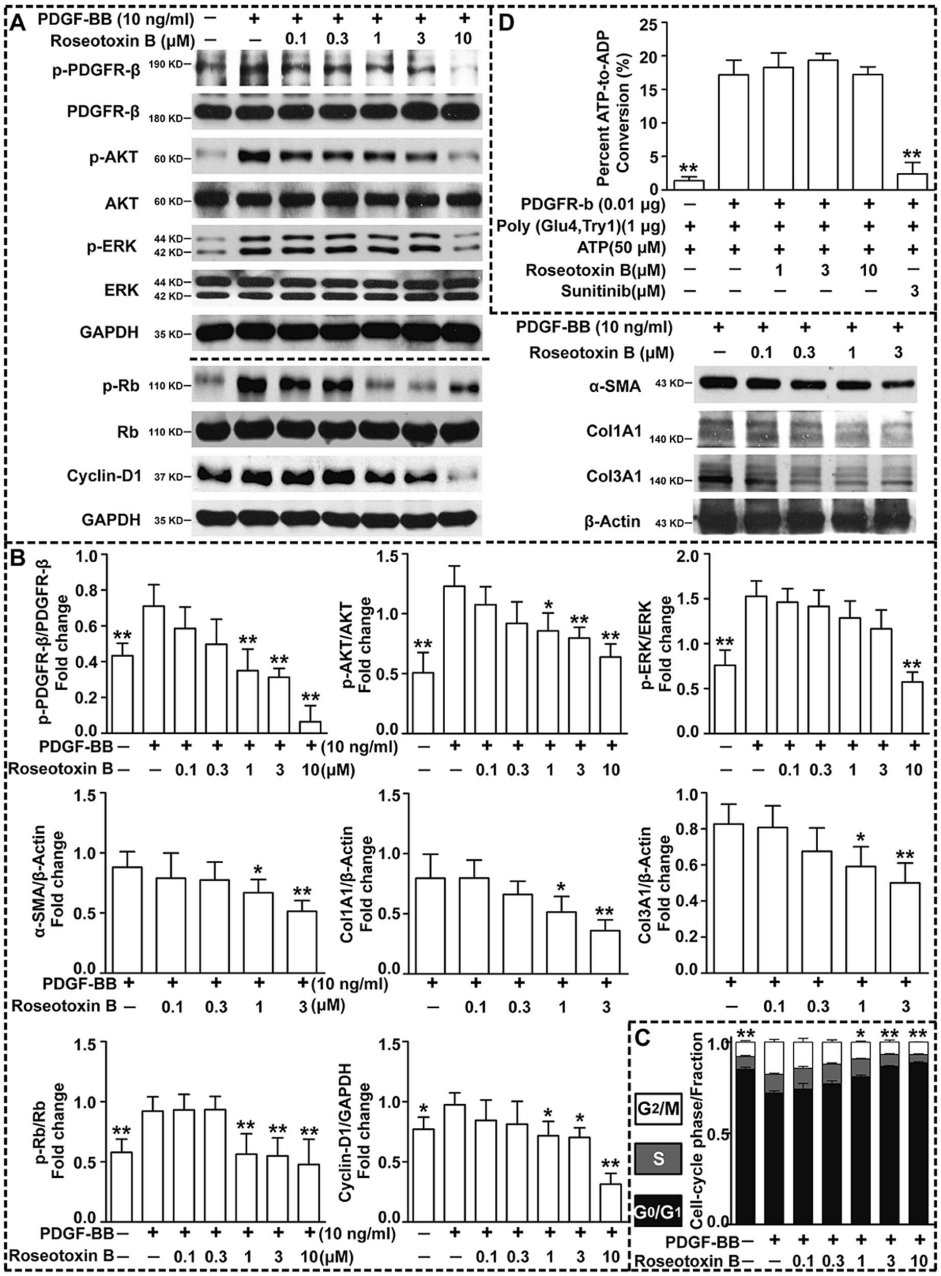
PDGF-B/PDGFR-β pathway was inhibited by roseotoxin B. **a**, **b** Representative Western blotting bands and their data summaries of the expression of proteins in PDGF-B/PDGFR-β pathway. Stimulation of CFSC-8B cells with 10 ng/ml of PDGF-BB followed by the detection of the expression of proteins by Western blotting. The Western blotting data are presented as means ± SEM. **c** Roseotoxin B led to cell cycle arrest of LX-2 cells under PDGF-BB stimulation. LX-2 cells were incubated with 10 ng/ml PDGF-BB in the absence or presence of roseotoxin B (0.1–10 μM) for 48 h. Roseotoxin B (1–10 μM) could reduce the proportion of LX-2 cells distributed in S and G_2_-M phases while led to the accumulation of LX-2 cells in G_0_-G_1_ phase. **d** Effect of roseotoxin B on the catalytic activity of human PDGFR-β was determined using PDGFR-β activity assay kit (*n* = 5 each group). All data represent means ± SEM of three independent experiments performed in triplicate. ^*^P < 0.05, ^**^P < 0.01 versus the control group.

There is sufficient scientific evidence that activated HSCs are the key target cells in liver fibrosis^8,9^. To assess the influences of roseotoxin B on PDGF-B/PDGFR-β pathway, the expression levels of p-PDGFR-β/PDGFR-β, p-AKT/AKT, p-ERK/ERK, p-Rb/Rb, and Cyclin-D1 were examined in CFSC-8B cells. Stimulation of CFSC-8B cells with PDGF-BB (10 ng/ml) led to the increased expression level of p-PDGFR-β, p-AKT, p-ERK, p-Rb, and Cyclin-D1 (Fig. 6a, b). However, roseotoxin B dose-dependently decreased their levels in PDGF-BB-activated CFSC-8B cells (Fig. 6a, b). Additionally, roseotoxin B also reduced the expression level of liver fibrosis-related α-SMA, Col1A1 and Col3A1 in the PDGF-BB-activated CFSC-8B cells (Fig. 6a, b). Moreover, PDGF-BB promoted cell cycle progression in LX-2 cells, but inhibited by roseotoxin B in a concentration-dependent manner (Fig. 6c). Notably, roseotoxin B did not display an inhibitory effect on the kinase activity of PDGFR-β (Fig. 6d), suggesting it was unable to bind to the intracellular tyrosine kinase domain of PDGFR-β.

### Roseotoxin B suppressed PDGF-BB-induced HSCs activation, proliferation, and migration

**Fig. 7:**
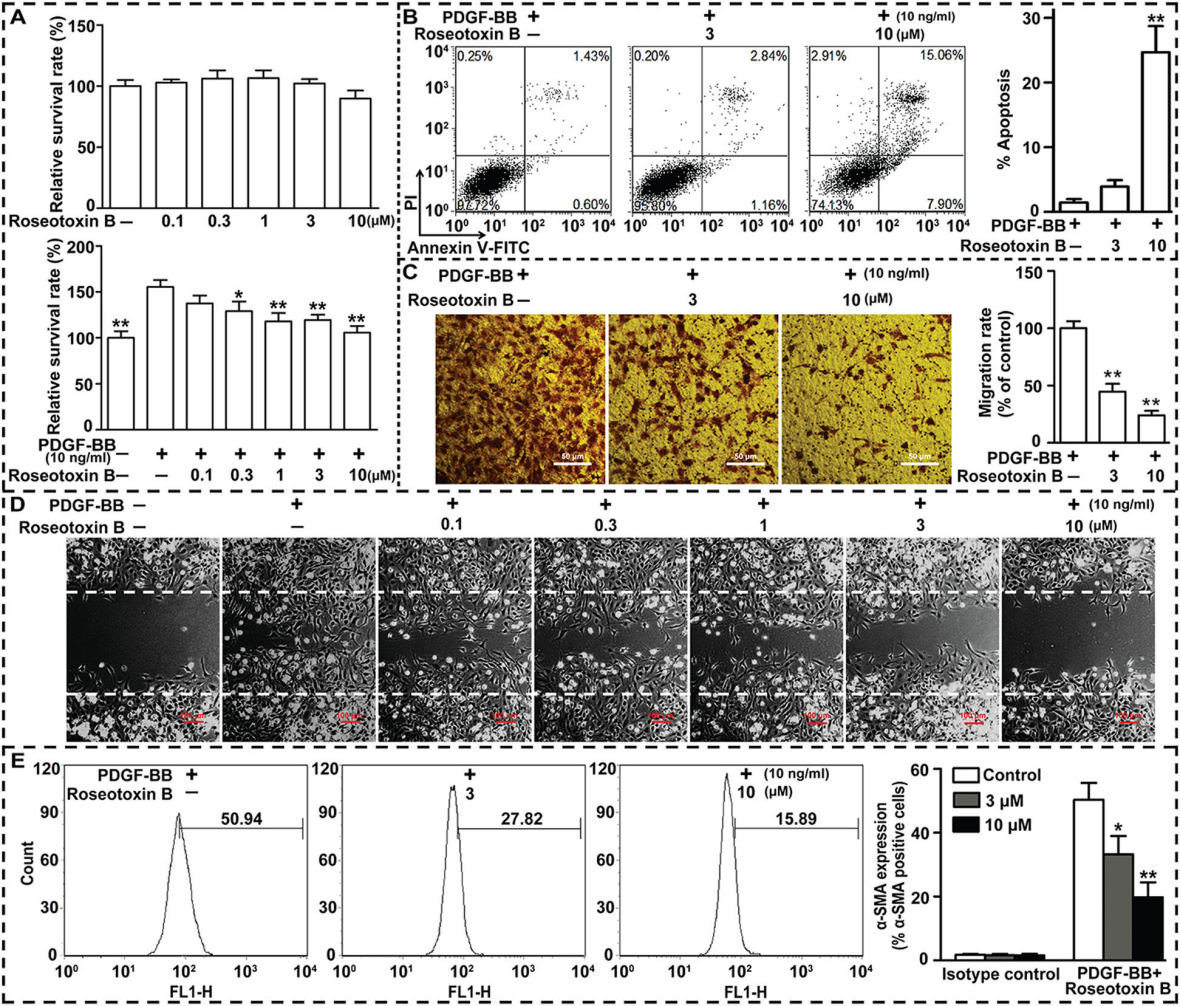
Roseotoxin B inhibited the PDGF-BB-induced HSC activation, survival, migration, and proliferation in vitro. **a** The effects of roseotoxin B on the survival and proliferation of quiescent LX-2 cells and PDGF-BB-activated LX-2 cells were assessed with MTT assay (*n* = 8 each group). **b** Roseotoxin B induced LX-2 cells apoptosis under PDGF-BB treatment (*n* = 4 each group). **c**, **d**) The inhibitory effects of roseotoxin B on PDGF-BB-induced CFSC-8B cells migration were evaluated according to the transwell invasion assays (scale bar = 50 μm) and the linear wound repair capacity (scale bar = 100 μm). **e** Expression of α-SMA, a HSCs activation marker, was significantly suppressed by roseotoxin B. After roseotoxin B treatment for 48 h, the percentage of α-SMA-positive HSCs on gated PDGF-BB-induced active LX-2 cells was significantly reduced. The data are expressed as histograms illustrating means ± SEM of three independent experiments. ^*^P < 0.05, ^**^P < 0.01.

As shown in Fig. 7a, roseotoxin B barely inhibited LX-2 cells survival without PDGF-BB stimulation. Notably, roseotoxin B concentration-dependently suppressed the proliferation of PDGF-BB-activated LX-2 cells. Additionally, the percentage of apoptotic cells in PDGF-BB-activated LX-2 cells was increased by roseotoxin B (Fig. 7b). PDGF-BB stimulation could enhance the migration and wound healing ability of CFSC-8B cells (Fig. 7c, d), whereas the PDGF-BB-induced CFSC-8B cells migration and wound repair were inhibited by roseotoxin B (Fig. 7c, d). To evaluate the impact of roseotoxin B on HSC activation induced by PDGF-BB, intracellular expression of α-SMA, the HSC activation marker, was examined by flow cytometry. The percentage of α-SMA positive LX-2 cells was significantly decreased after roseotoxin B treatment (Fig. 7e). Of note, these pharmacological effects of roseotoxin B on HSCs have been mutually verified in the rat HSC line CFSC-8B cells and the immortalized human HSC line LX-2 cells (Figs. 6 and 7 and Supplementary Fig. 4).

### Roseotoxin B blocked PDGF-B/PDGFR-β pathway in vitro and in vivo through disrupting the assembly of PDGF-BB/PDGFR-ββ complex

How did roseotoxin B regulate PDGFR-β to exert its inhibitory effect on PDGF-B/PDGFR-β pathway in HSCs? Computational molecular docking revealed that roseotoxin B might occupy the D2 domain of PDGFR-β, and the central feature of their interface is a large and hydrophobic cluster, formed by the side chains of Ile137, Phe138, Leu139, Ile145, Pro148, Val297 from PDGFR-β-D2 and the hydrophobic groups from roseotoxin B (Supplementary Fig. 5A, B). Additionally, the side chains of Glu133, Glu134, Phe136, Thr140, Glu141, Thr143, Glu144, Thr146, and Leu209 from PDGFR-β-D2 might interact with roseotoxin B by van der Waals forces. Notably, Phe138 of PDGFR-β-D2 might also form hydrogen bond interactions, hydrophobic force, and van der Waals interactions with roseotoxin B (Supplementary Fig. 5B, C), suggesting that the Phe138 is likely to contribute a great deal to the formation of roseotoxin B/PDGFR-β. Taken together, these observations demonstrated that the roseotoxin B conformation, rather than the point-to-point contacts at their interface, was the structural determinant for roseotoxin B/PDGFR-β binding.

**Fig. 8:**
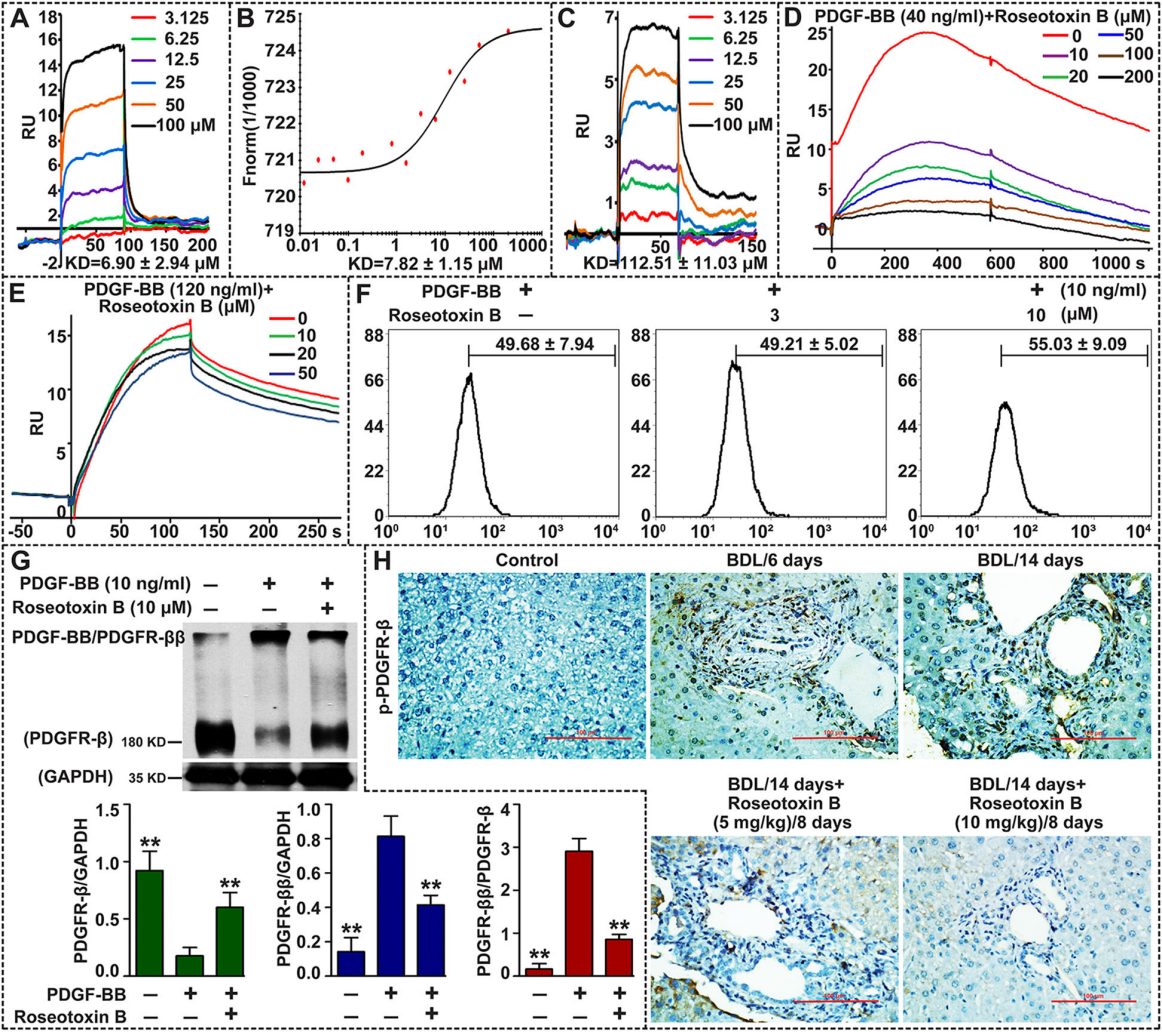
Roseotoxin B inhibited the PDGF-B/PDGFR-β pathway through disrupting the PDGF-BB/PDGFR-ββ complex formation. The interaction between roseotoxin B and human PDGFR-β (Met 1-Lys 531) protein was measured by SPR analysis (**a**) and microscale thermophoresis technology (**b**). **c** The binding ability of roseotoxin B to human PDGFR-β (Met 1-Lys 531) with Phe138/Ala138 mutation was measured by the SPR analysis. **d**, **e** Roseotoxin B disrupted the interaction of PDGF-BB/PDGFR-β, which was measured by the SPR analysis. **f** Roseotoxin B barely affected the surface expression of PDGFR-β in HSCs. HSCs were treated with PDGF-BB (10 ng/ml) in the presence of roseotoxin B for 24 h, then the surface expression of PDGFR-β in HSCs was detected by flow cytometry. **g** Roseotoxin B disrupted the assembly of PDGF-BB/PDGFR-ββ complex in HSCs, which was measured by non-denaturing Western blotting. The data are expressed as histograms illustrating means ± SEM of three independent experiments. ^*^P < 0.05, ^**^P < 0.01. **h** The inhibitory effect of roseotoxin B on the expression of p-PDGFR-β in the livers of mice with liver fibrosis induced by BDL; hepatic immunohistochemical staining with p-PDGFR-β antibody; the data represented here represent one of three independent experiments with similar results.

To validate the molecular docking analysis, we used surface plasmon resonance biosensor (SPR-Biacore T200) which is a straightforward and novel technology for to study intermolecular interactions to examine whether the extracellular segment of PDGFR-β contained a binding area for roseotoxin B (Fig. 8a–e). As shown in Fig. 8a, the responses observed for roseotoxin B correlated with its concentrations, and the SPR yielded an affinity for the roseotoxin B/PDGFR-β (Met1-Lys 531) interaction of 6.90 ± 2.94 μM. Additionally, the interaction of roseotoxin B/PDGFR-β (Met1-Lys 531) were also confirmed by microscale thermophoresis technology (MST), which gave a Kd value of 7.82 ± 1.15 μM (Fig. 8b). Furthermore, we constructed a series of mutation on amino acids to identify the specific location in the extracellular segment of human PDGFR-β (Met1-Lys 531) to which roseotoxin B bound. We filtered the binding affinities of roseotoxin B to each mutated PDGFR-β protein using the SPR, and the Kd value of Phe138/Ala138 mutated group was 112.51 ± 11.03 μM, which was approximately 16-fold higher than that of the wild-type group (Fig. 8c). This result not only confirmed the contribution of Phe138 to roseotoxin B/PDGFR-β interaction, but also indicated that the D2 domain of PDGFR-β was their binding area. What would happen when roseotoxin B was combined with PDGFR-β-D2? As shown in Fig. 8d, e, the SPR response values were significantly decreased by roseotoxin B in a dose-dependent manner compared with the 40 ng/ml PDGF-BB group, but the inhibitory effect of roseotoxin B on PDGF-B/PDGFR-β interaction was obviously weakened by 120 ng/ml PDGF-BB, which strongly suggested that the assembly of PDGF-B/PDGFR-β complex was disrupted by roseotoxin B. Accordingly, the computational analysis had been verified experimentally.

Whether roseotoxin B affected the assembly of PDGF-BB/PDGFR-ββ complex in HSCs. As shown in Fig. 8f, roseotoxin B scarcely affected PDGFR-β expression on HSCs membrane, as measured by flow cytometry. When the ligand PDGF-B binds to its receptor PDGFR-β, it can induce the formation of PDGF-BB/PDGFR-ββ complex, leading to HSCs survival, proliferation, transdifferentiation, migration, and extracellular matrix production^15^. However, the PDGF-BB-induced PDGF-BB/PDGFR-ββ complex in cultured HSCs was significantly blocked by roseotoxin B, as measured by non-denaturing Western blotting (Fig. 8g). Meaningfully, immunohistochemical examination demonstrated that the expression level of p-PDGFR-β was significantly increased in BDL-induced fibrotic lesions, whereas the level of p-PDGFR-β in vivo was obviously reduced by therapeutic treatment of roseotoxin B (Fig. 8h).

## Discussion

In this study, roseotoxin B was found to be able to serve as a candidate agent for liver fibrosis. This conclusion was reached based on the following observations: first, roseotoxin B can effectively attenuate cholestasis-induced liver damage and collagen fiber formation in murine livers; second, most patients with liver fibrosis show high level of p-PDGFR-β, the marker of activated PDGF-B/PDGFR-β pathway, in their liver samples, suggesting that the PDGF-B/PDGFR-β pathway is an important therapeutic target for liver fibrosis; third, roseotoxin B can inhibit the fibrogenesis of HSCs by blocking the PDGF-B/PDGFR-β pathway; finally, the assembly of PDGF-BB/PDGFR-ββ complex in HSCs was disrupted by roseotoxin B.

Bile duct ligation (BDL)-induced murine liver fibrosis is one of the most widely used animal models for identifying anti-fibrotic drugs^24,25^. Using this model, roseotoxin B was found to have anti-fibrosis characteristics against the cholestasis-induced liver fibrosis. The experimental results indicated that roseotoxin B could significantly relieve the extent of hepatocyte injury and the degree of cholestatic fibrosis caused by BDL. It is well known that pathologic cellular apoptosis is an important nexus of liver injury and fibrosis^22,26^. Generally, the cellular apoptosis is the first pathologic response to liver injury^22,27^. However, the dysregulated apoptosis can disrupt the integrity of hepatocytes and aggravate hepatic inflammation^22,28–30^. If the hepatic inflammation continues to deteriorate, it will cause quiescent HSCs become into activated HSCs leading to liver fibrosis^9,31^. To make matters worse, the activated HSCs can in turn exacerbate hepatocyte cell death and hepatic inflammation^22^. Notably, the number of cellular apoptosis in non-fibrotic regions of liver tissues was significantly reduced by roseotoxin B. We accordingly believed that roseotoxin B was likely to have the ability to calm the liver injury and fibrosis caused by cholestatic stress.

Subsequently, we found that the protein thermal stability of PDGFR-β in cholestatic fibrotic liver tissue was protected by roseotoxin B, which was mainly due to ligand-induced thermal stabilization of target protein, suggesting that the PDGFR-β might be a direct target of roseotoxin B. Very importantly, human tissue microarrays showed that p-PDGFR-β expression level in about 80% of cirrhotic tissue and about 39% of hepatocellular carcinoma tissue was much higher than those of normal livers, indicating that p-PDGFR-β could represent an important hallmark and therapeutic target for chronic liver disease, especially the liver fibrosis. Therefore, we were eager to explore how roseotoxin B inhibited PDGFR-β activation and subsequently exhibited its anti-fibrotic effect on liver fibrosis. It is now very clear that the pathological environment of cholestatic liver injury prompts a large number of PDGFR-β expression in HSCs, and meanwhile a variety of liver resident cells are continuing to secrete a large amount of PDGF-BB. Notably, the PDGF-BB is not only a non-invasive marker in the diagnosis of liver fibrosis/cirrhosis but also the most effective in stimulating HSCs transdifferentiation and proliferation^15,32,33^. Several potential sources of myofibroblasts have been elucidated by rodent-based studies, demonstrating that 82–90% of myofibroblasts in livers with cholestasis-induced fibrosis were derived from HSCs^34^. The present study confirmed that roseotoxin B had the function of inhibiting PDGF-B/PDGFR-β pathway in HSCs, and the PDGF-BB-induced HSCs activation, proliferation, migration, cell cycle progression and collagen expression were all inhibited by roseotoxin B. It must however be noted that the inhibitory activity of roseotoxin B on PDGF-B/PDGFR-β was different from traditional tyrosine kinase inhibitor (TKI). Therefore, clarifying the underlying mechanism would have an important pharmacological significance. The TKI inhibits PDGFR-β through directly binding to the intracellular ATP pocket^15^. However, roseotoxin B caused the failure of PDGF-BB/PDGFR-β complex formation via directly binding to the D2 domain of PDGFR-β, thus blocking the PDGF-B/PDGFR-β pathway in HSCs and in vivo.

In conclusion, our study found a small molecule roseotoxin B which could play an anti-fibrosis role in cholestatic liver fibrosis through directly blocking the PDGF-B/PDGFR-β pathway in HSCs. Roseotoxin B would be a unique candidate agent for the treatment of liver fibrosis.

## Supplementary information

Supplementary Figure Legends

Suppl. Fig. 1

Suppl. Fig. 2

Suppl. Fig. 3

Suppl. Fig. 4

Suppl. Fig. 5

Suppl. Fig. 6
