# Supplementary material for: Roseotoxin B alleviates cholestatic liver fibrosis through inhibiting PDGF-B/PDGFR-β pathway in hepatic stellate cells
Source: Cell Death Dis. 2020 Jun 15;11(6):458. doi: 10.1038/s41419-020-2575-0 (PMC7296008; doi:10.1038/s41419-020-2575-0)
Supplement: Supplementary file 1 — Supplementary Figure Legends [file 41419_2020_2575_MOESM1_ESM.docx]

**Supplementary Figure Legends**

**Suppl. Fig. 1: The quantification of the fibrosis levels was performed according to Masson trichrome staining** (A)**, Sirius Red staining** (B)**, and immunohistochemical staining of α-SMA** (C)**.** Mean optical density (MOD), integrated optical density (IOD), MOD = IOD/area (sum). The data are expressed as histograms illustrating the means ± the SEM. of three independent experiments. ^*^*P* < 0.05, ^**^*P* < 0.01 versus the BDL/14 d group.

**Suppl. Fig. 2: The percentage of apoptotic cells in the non-fibrotic areas of liver tissues was measured according to TUNEL staining** (A), and the data are expressed as histograms illustrating the means ± the SEM. of three independent experiments, ^*^*P* < 0.05, ^**^*P* < 0.01 versus the BDL/14 d group; **the percentage of p-PDGFR-β positive cells in normal hepatic tissues, fibrotic areas of the fibrotic livers, and hepatocellular carcinoma tissues was assessed according to immunohistochemical staining of the p-PDGFR-β** (B), the data are expressed as histograms illustrating the means ± the SD. of these human samples, ^*^*P* < 0.05, ^**^*P* < 0.01 versus the normal hepatic tissues.

**Suppl. Fig. 3: Roseotoxin B targets the PDGFR-β protein in liver tissue with cholestatic liver fibrosis.** Total proteins extracted from murine liver tissue with cholestatic liver fibrosis were incubated with DMSO or roseotoxin B (10 μM) for 3 h, then they were subjected to CESTA. Subsequently, the protein solution was analyzed by SDS/PAGE electrophoresis and silver staining. Proteins with significant differences between the DMSO-incubated group and roseotoxin B-incubated group were obtained for mass spectrometric identification. As shown in the dashed boxes, PDGFR-β was the target protein that was protected by roseotoxin B-induced thermal stabilization.

**Suppl. Fig. 4: Roseotoxin B inhibited the PDGF-B/PDGFR-β pathway and suppressed PDGF-BB-induced HSC survival, proliferation, and migration *in vitro*.** (A) The effects of roseotoxin B on the survival and proliferation of PDGF-BB-activated CFSC-8B cells were assessed with MTT assay (n = 8 each group). (B) The inhibitory effect of roseotoxin B on PDGF-BB-induced LX-2 cells migration were evaluated according to the transwell invasion assays (scale bar = 100 μm). (C and D) Stimulation of LX-2 cells with 10 ng/ml of PDGF-BB followed by the detection of the expression of proteins by Western blotting. Representative Western blotting bands and their data summaries of the expression of proteins in PDGF-B/PDGFR-β pathway (p-PDGFR-β, PDGFR-β, p-AKT, and AKT), cell cycle associated protein cyclin-D1, and apoptosis related protein caspase-3. All data represent the means ± SEM. of three independent experiments performed in triplicate. ^*^*P* < 0.05, ^**^*P* < 0.01 versus the control group.

**Suppl. Fig. 5: Structure model of PDGFR-β-roseotoxin B.** (A) The computational binding mode of roseotoxin B in human PDGFR-β-D2 was established using the AutoDock 4.2 software, and the model was generated in Accelrys Discovery Studio software. (B and C) Molecular docking analysis of roseotoxin B and PDGFR-β.

**Suppl. Fig. 6: Schematic diagram of the mechanism underlying the anti-fibrotic effect of roseotoxin B on cholestatic liver fibrosis by targeting PDGFR-β to block the PDGF-B/PDGFR-β pathway in HSCs.**
