# Supplementary figures and images for: Roseotoxin B alleviates cholestatic liver fibrosis through inhibiting PDGF-B/PDGFR-β pathway in hepatic stellate cells
Source: Cell Death Dis. 2020 Jun 15;11(6):458. doi: 10.1038/s41419-020-2575-0 (PMC7296008; doi:10.1038/s41419-020-2575-0)

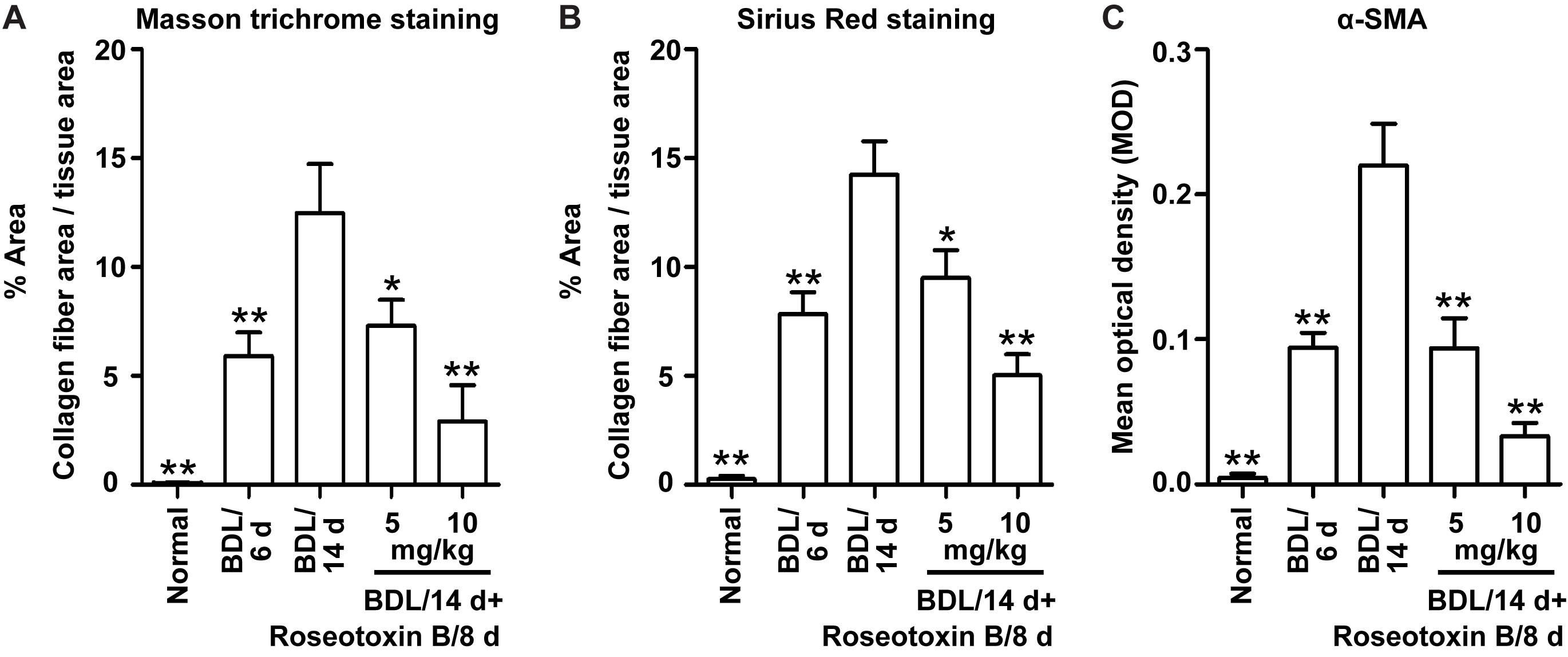

Supplement: Supplementary file 2 — Suppl. Fig. 1 [file 41419_2020_2575_MOESM2_ESM.tif]

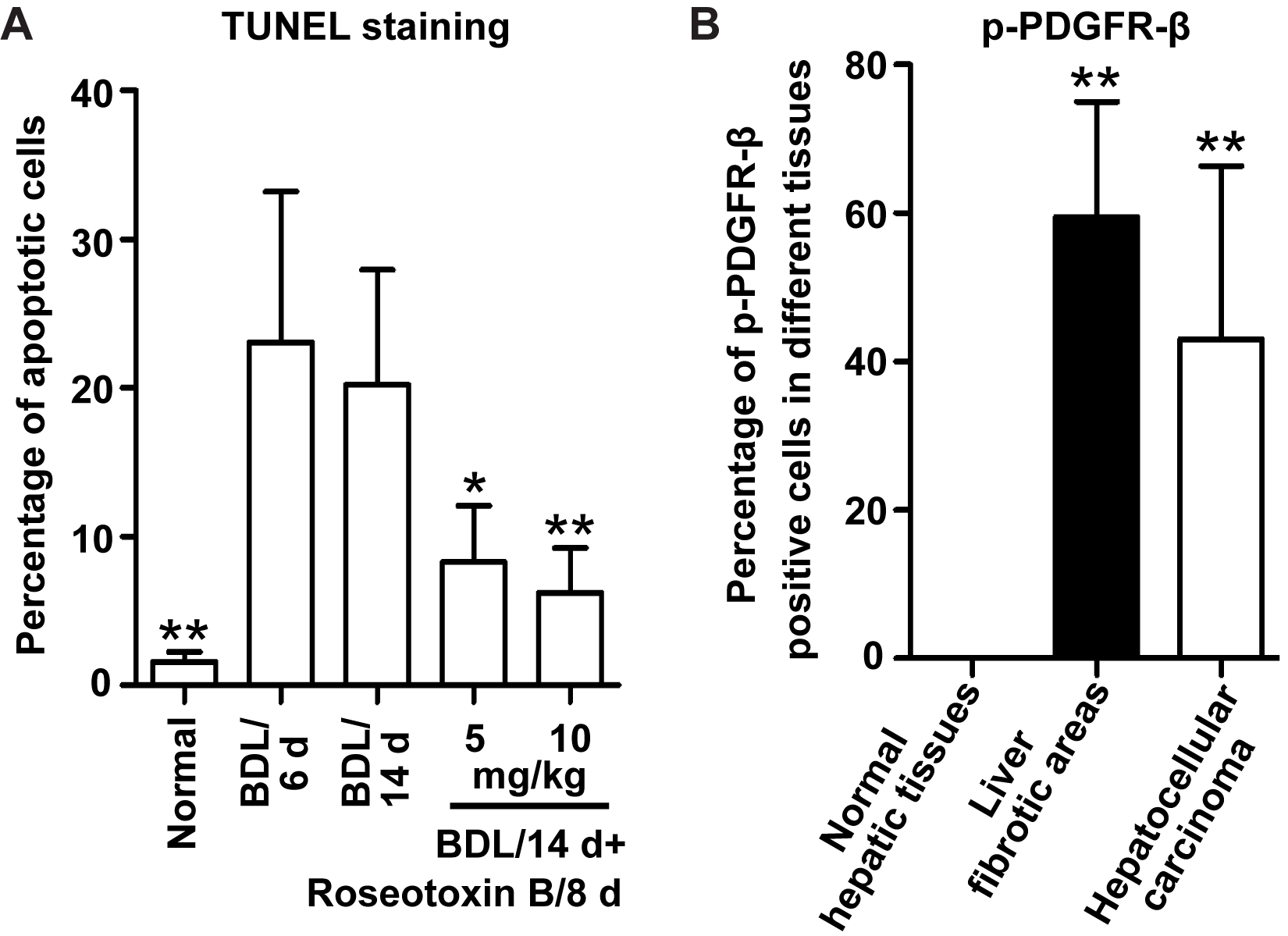

Supplement: Supplementary file 3 — Suppl. Fig. 2 [file 41419_2020_2575_MOESM3_ESM.tif]

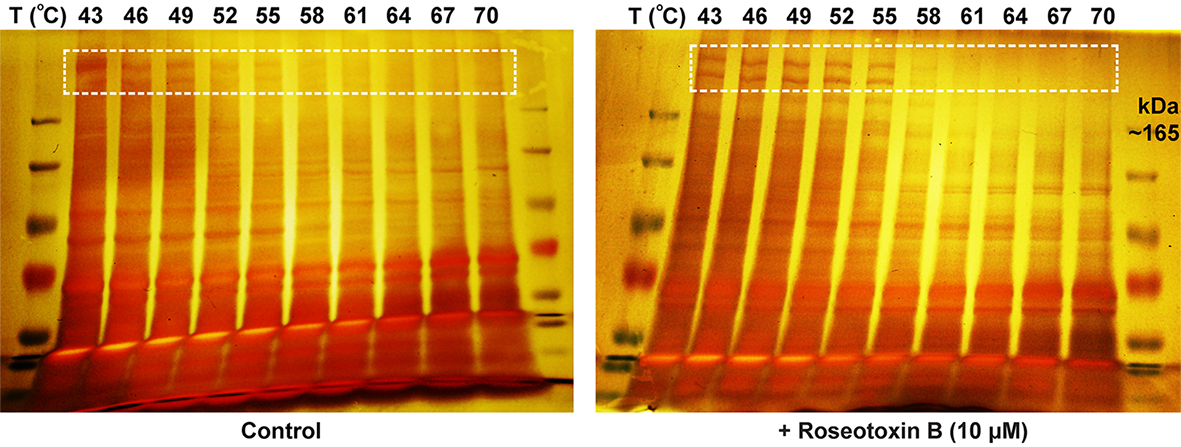

Supplement: Supplementary file 4 — Suppl. Fig. 3 [file 41419_2020_2575_MOESM4_ESM.tif]

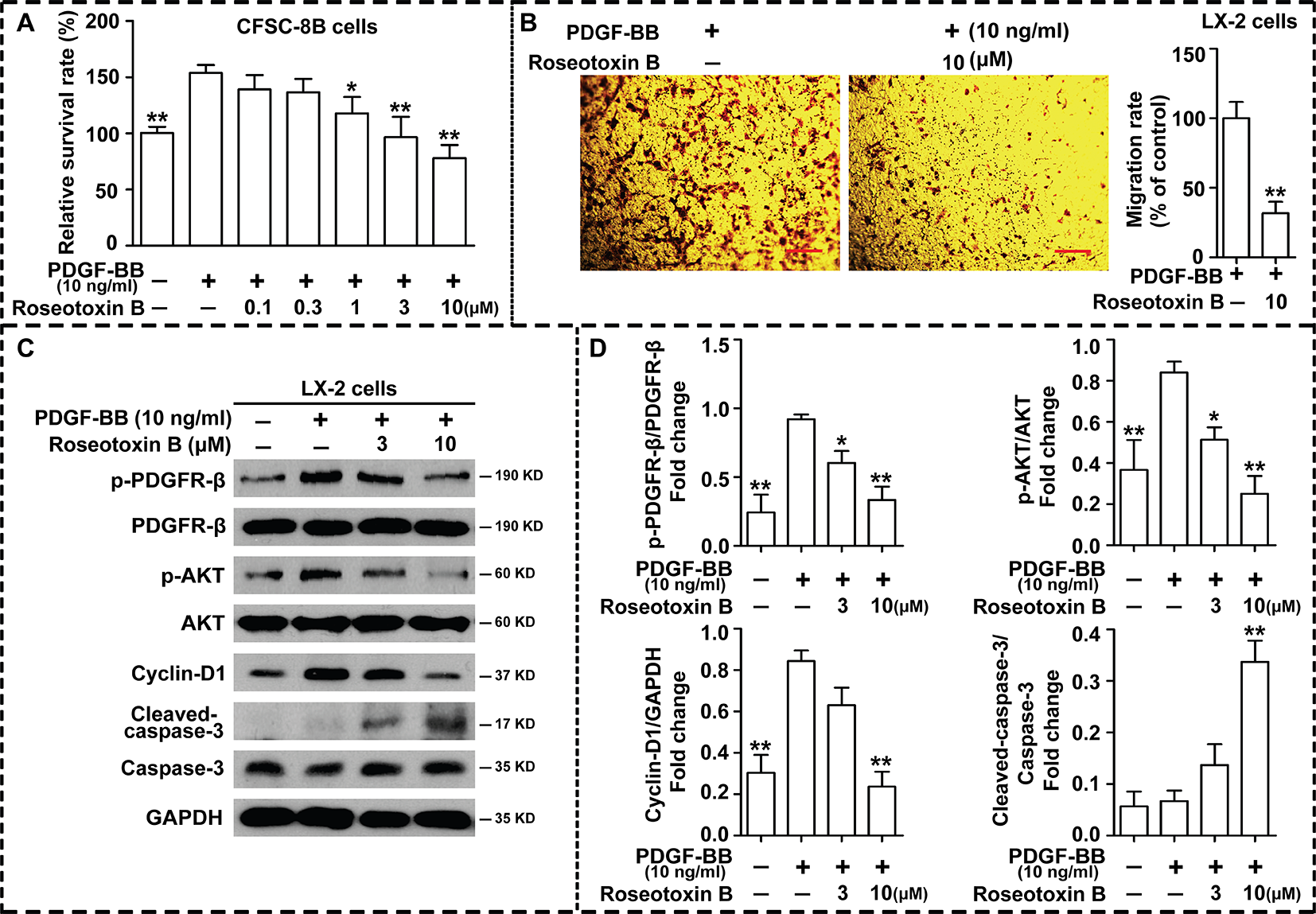

Supplement: Supplementary file 5 — Suppl. Fig. 4 [file 41419_2020_2575_MOESM5_ESM.tif]

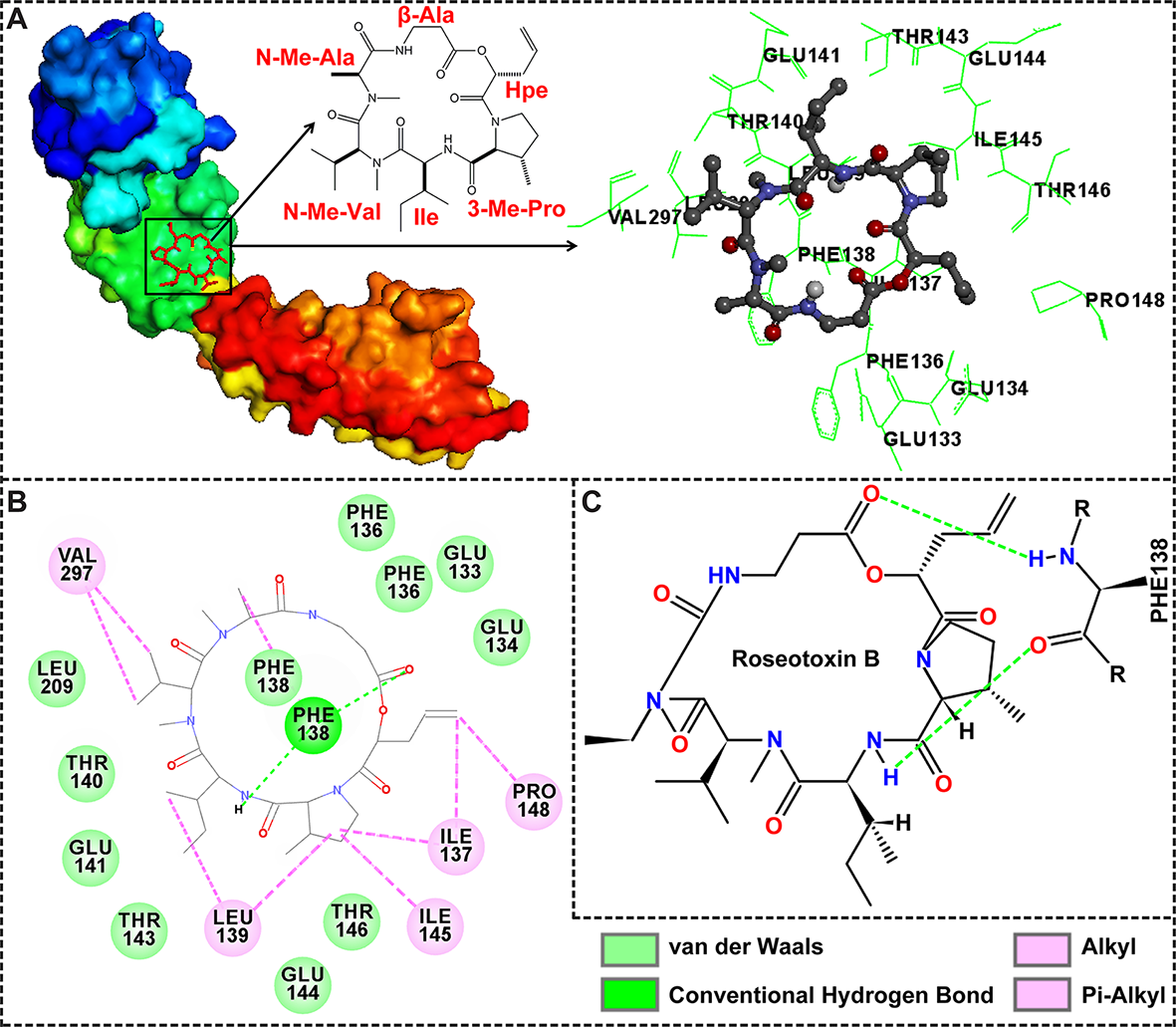

Supplement: Supplementary file 6 — Suppl. Fig. 5 [file 41419_2020_2575_MOESM6_ESM.tif]

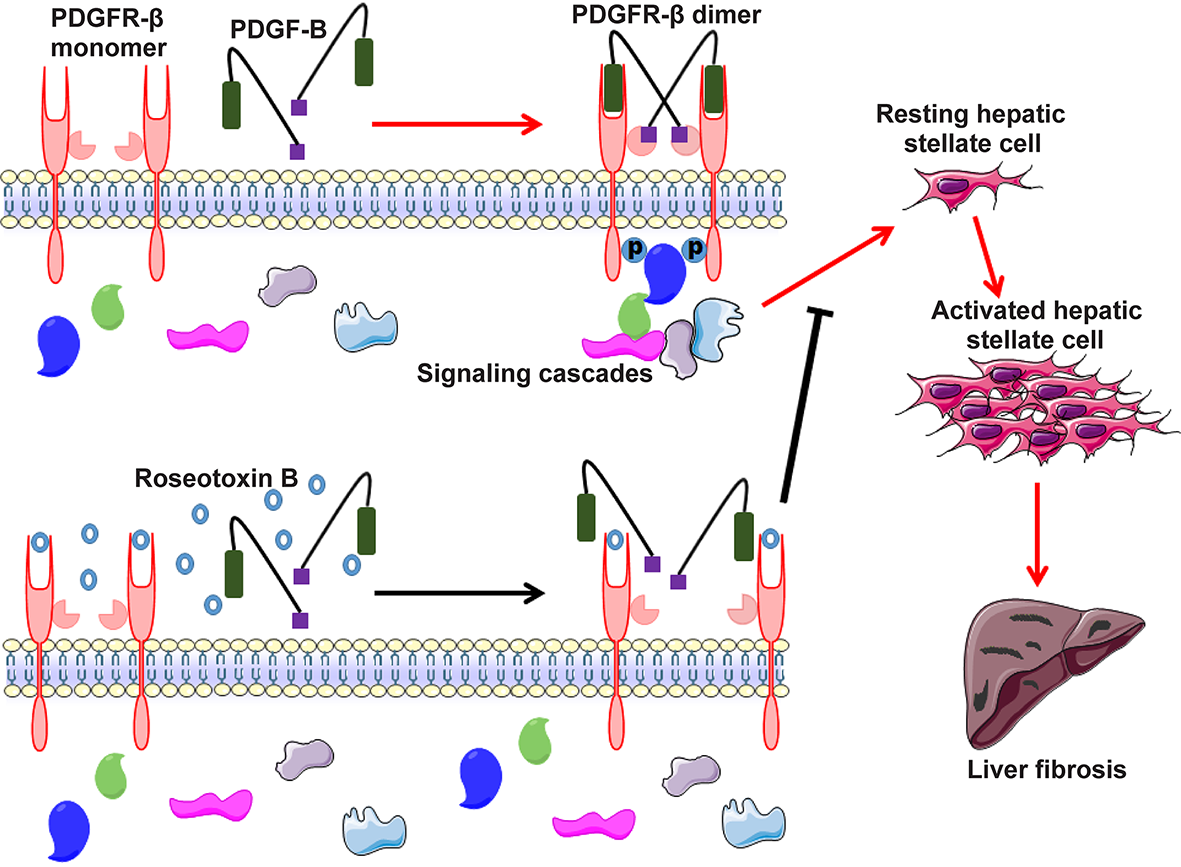

Supplement: Supplementary file 7 — Suppl. Fig. 6 [file 41419_2020_2575_MOESM7_ESM.tif]
